# Comparative Proteome-Wide Abundance Profiling of Yeast Strains Deleted for Cdc48 Adaptors

**DOI:** 10.3390/proteomes12040031

**Published:** 2024-10-30

**Authors:** Valentina Rossio, Joao A. Paulo

**Affiliations:** Department of Cell Biology, Harvard Medical School, Boston, MA 02115, USA

**Keywords:** TMT proteomics, Ubiquitin Proteasome System, *S. cerevisiae*, Cdc48, proteome, UBX domain-containing proteins

## Abstract

The yeast ATPase Cdc48 (known as p97/VCP in human cells) plays an important role in the Ubiquitin Proteasome System. VCP is essential for cancer cell proliferation, and its dysregulation has been implicated in several neurodegenerative diseases. Cdc48 functions by extracting ubiquitylated proteins from membranes, protein complexes and chromatin by often facilitating their proteasomal degradation. Specific adaptors or cofactors, primarily belonging to the UBX domain-containing protein family (which has seven members in *Saccharomyces cerevisiae*) recruit Cdc48 to ubiquitylated proteins. Here, we employed sample multiplexing-based quantitative mass spectrometry to profile global protein abundance in p97 adaptor deletion strains, specifically comparing seven single deletion strains of UBX domain-containing proteins and the Cuz1 deletion strain, which belongs to the zinc finger AN1-type domain protein family. We observed that each strain showed unique sets of differentially abundant proteins compared to the wild type. Our analysis also revealed a role for *Ubx3* in maintaining wild type levels of mitochondrial proteins. Overall, we identified ~1400 differentially abundant proteins in the absence of a specific Cdc48 adaptor. This unique dataset offers a valuable resource for studying the functions of these adaptors, aiming to achieve a better understanding of the cellular processes regulated by Cdc48 itself and to deepen our understanding of the Ubiquitin Proteasome System.

## 1. Introduction

The Ubiquitin Proteasome System (UPS) is a highly conserved network of enzymes that regulate many cellular functions by attaching ubiquitin to substrates [1,2,3,4]. While the best characterized function of ubiquitylation is targeting proteins for proteasomal degradation [5,6], ubiquitylation also has non-degradative roles [7,8], such as altering protein activity and localization.

In *S. cerevisiae*, ATPase Cdc48, known as p97/VCP in mammalian cells, is an important player of the UPS, and its gene is essential. Mutations in human p97 have been linked to neurodegenerative diseases [9,10,11] and have been associated with an inherited genetic disease (VCP disease) [12,13]. Furthermore, p97 is upregulated in many types of cancers, making it a potential target for anti-cancer therapeutics [14,15,16]. In fact, specific p97 inhibitors have been shown to suppress cancer progression and are currently under clinical trials [15,17]. Cdc48 acts by extracting ubiquitylated proteins from chromatin, membranes or macromolecular complexes often to facilitate their proteasomal degradation [11,18,19,20]. Additionally, Cdc48 has been shown to unfold well-folded proteins to ensure their proteasomal degradation [21]. Cdc48 is indeed involved in many cellular processes, including membrane organelle fusion [22], protein complex assembly and disassembly [23], clearance of protein aggregates [24], endoplasmic reticulum-associated protein degradation (ERAD) [25,26] and mitotic spindle disassembly [27].

The different functions of Cdc48 are dictated by interchangeable cofactors or adaptors, which determine its substrate specificity by recruiting Cdc48 to specific ubiquitylated proteins [11]. One prominent family of Cdc48 adaptors is characterized by the presence of the ubiquitin regulatory X (UBX) domain, responsible for binding to Cdc48 [28,29]. In *S. cerevisiae*, this family includes seven members (*Ubx1*–*Ubx7*), whereas human cells have thirteen [29]. Apart from the UBX domain-containing proteins, other Cdc48 cofactors lacking the UBX domain have also been discovered. One such example is Cuz1 (known as Zfand1 in mammalian cells), a member of the zinc finger AN1-type domain family of proteins [30]. Cuz1 interacts directly with both Cdc48 and the proteasome and protects cells from arsenic-induced toxicity [31,32]. The same protective role has been observed for the homolog Zfand1 [33].

A previous study identified substrates of the UBX adaptor proteins by profiling the ubiquitylated proteins changing in yeast deletion strains of single adaptor genes using a proteomic approach [34]. This study identified ubiquitylated proteins increasing in specific mutants compared to wild type. This was expected since, in these mutants, the adaptors connecting Cdc48 to ubiquitylated proteins are missing. As a result, these proteins accumulate, because they are not being delivered to the proteasome for degradation. Unexpectedly, a considerable number of ubiquitylated proteins were also found to decrease in these mutants as well. The decrease in ubiquitylated proteins could be due to indirect effects of deleting UBX proteins or because UBX proteins can protect these proteins from deubiquitylation [35]. Interesting, a global comparison of proteomic changes in the absence of specific Cdc48 adaptors has not yet been performed in *S. cerevisiae*. Such analysis could help pinpoint the cellular processes regulated by these adaptors and provide novel insights into how broadly the Cdc48 network influences cellular functions.

Here, we employed sample multiplexing-based quantitative mass spectrometry to globally profile and compare protein abundance on a proteome-wide scale across individual yeast deletion strains lacking each of the seven UBX proteins (*Ubx1*–*Ubx7*) and Cuz1 in a single multiplexed experiment. We observed that these adaptors have different impacts on the global proteome: notably, a strong effect was measured in the *UBX1* (*SHP1*), *CUZ1* and *UBX3* deletion strains, while the deletion of other adaptors, like *UBX4*, *UBX5* and *UBX6*, had only a minor effect. The deletion of each adaptor induced a specific set of differentially abundant proteins, either up- or downregulated, suggesting that they influence specific and distinct cellular functions. For example, our analysis showed that only *SHP1* deletion increased the protein level of Rpn4, a transcription factor that is involved in the response to proteotoxic stress. Coincidently, in the *shp1Δ* strain, the downregulation of ribosomal subunits was observed, which is also known to be an effective response to proteotoxic stress. This suggests that the UPS is likely overwhelmed in *shp1Δ* cells. Furthermore, the specific downregulation of mitochondrial proteins was detected in the absence of *UBX3*, suggesting that this adaptor could be important to ensure their wild type level. Our extensive dataset of ~1400 differentially abundant proteins in single adaptor deletion strains offers a valuable resource for studying these adaptors and for a better understanding of the cellular processes regulated by them and by Cdc48 itself.

## 2. Materials and Methods

### 2.1. Materials

The materials used in this study were purchased from different sources. The *S. cerevisiae* BY4742 wildtype strain and the strains carrying single deletions of p97 adaptors were from Open Biosystems in Huntsville, AL, USA. YPD media was obtained from Sunrise Science in San Diego, CA, USA. Protease inhibitors, the BCA protein quantification kit, the Tandem Mass Tag (TMTpro) reagents and trypsin were all from Thermo Fisher Scientific in Rockford, IL, USA. Lys-C protease was acquired from Fujifilm Wako in Richmond, VA, USA. Mass spectrometry-grade water and organic solvents were obtained from J.T.Baker in Center Valley, PA, USA. StageTip Empore-C18 disks were supplied from CDSanalytical in Oxford, PA, USA, while Sep-Pak cartridges (50 mg) were acquired from Waters Milford, MA, USA.

### 2.2. Yeast Strains, Growth Conditions and Protein Extraction

Our starter cultures were from a patch of yeast cells (rather than single colonies) on a plate streaked out from our original (purchased) stock to ensure that the effects of secondary adaptive mutations in single cells were likely diluted in the larger population of cells. Yeast cultures were grown at 25 °C overnight in YEPD medium containing 1% yeast extract, 2% bactopeptone and 2% glucose. The following day, the cultures were diluted with fresh YEPD medium to an OD of 0.2 (*shp1Δ* cells grew slower and were diluted to OD of 0.35). The cultures were then incubated at 25 °C until they reached the mid-exponential growth phase, corresponding to an OD of 1. Cells were harvested by centrifugation (2000× *g* for 2 min), and the resulting cell pellet was rinsed with 1 mL of sterile water. Cell pellets were immediately flash-frozen in liquid nitrogen and stored at −80 °C. We performed cell lysis and protein precipitation as described previously [36]. Cell pellets were resuspended in a buffer solution (lysis buffer) containing 8 M urea in 200 mM EPPS (pH 8.5) and supplemented with protease inhibitors. Cells were subjected to mechanical disruption with a beat beater at 4 °C. The protein concentration was quantified by performing a BCA assay, according to the manufacturer’s protocol. Proteins were treated with 5 mM tris(2-carboxyethyl)phosphine (TCEP) for 20 min to reduce them. After reduction, proteins were alkylated with 10 mM iodoacetamide for 20 min (in darkness), and the reaction was stopped by the addition of 10 mM dithiothreitol (DTT) (in darkness). Chloroform–methanol precipitation was performed on 100 µg of protein from each sample [37].

### 2.3. Protein Digestion, TMT Labeling and Sample Processing

**Figure 1:**
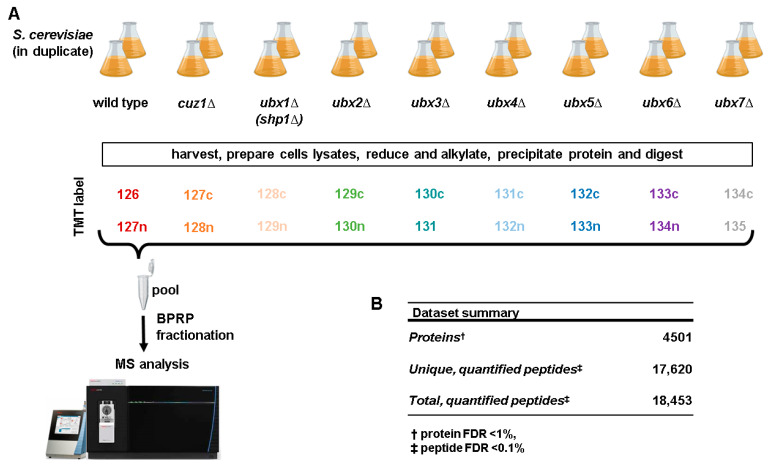
Experimental workflow and summary of the dataset. (**A**) Workflow of the TMTpro18-plex experiment highlighting the yeast strains used, the sample order, the labeling strategy and the LC-FAIMS-MS/MS analysis. Briefly, cells were harvested, lysed and proteins were extracted and digested with Lys-C, followed by trypsin. The resulting peptides were labeled with tandem mass tag (TMTpro) reagents, as indicated, pooled 1:1 and fractionated by basic pH reversed-phase (BPRP) HPLC prior to mass spectrometry (MS) analysis. This panel has been assembled, in part, using Biorender.com. (**B**) Summary of the dataset.

For protein digestion, 1 µg of Lys-C was used overnight at 24 °C, followed by 1 µg trypsin for 6 h at 37 °C per 100 µg of protein. A final volume of 30% acetonitrile was added to the digested peptides. Each sample, containing 50 µg of peptides, was labeled with 100 µg of the appropriate TMTpro labeling reagent, as indicated in Figure 1A (wild type duplicates 126, 127n; *cuz1Δ* duplicates: *127c 128n*; *shp1Δ* (*ubx1Δ*) duplicates: *128c 129n*; *ubx2Δ* duplicates: *129c 130n*; *ubx3Δ* duplicates: *130c 131*; *ubx4Δ* duplicates: 131c 132n; *ubx5Δ* duplicates: *132c 133n*; *ubx6Δ* duplicates *133c 133n*; *ubx7Δ* duplicates: *134c 135*). The labeling reaction proceeded for one hour at room temperature. Before continuing sample preparation, we assessed the labeling efficiency: ~1 µg of digested peptides were combined from each sample and desalted using StageTip [38], which was analyzed to verify that labeling efficiency was greater than >97% [39,40]. Hydroxylamine was then added to each sample to a final concentration of approximately 0.3% to quench the labeling reaction, followed by a 15 min incubation at room temperature. Finally, all the samples were mixed in equal proportion (1:1) and desalted using a 50 mg Sep-Pak solid-phase extraction column. Fractionation was executed with basic pH reversed-phase (BPRP) HPLC [41].

An Agilent 1260 pump (Lexington, MA, USA) equipped with an Agilent 300 Extend C18 column (3.5 μm particles, 2.1 mm ID, 250 mm length) was utilized. Peptides were fractionated using a 50-min linear gradient from 5% to 35% acetonitrile in 10 mM ammonium bicarbonate (pH 8) at a flow rate of 0.25 mL/min. A total of 96 fractions were collected, concatenated and condensed into 24 superfractions, resulting in two sets of 12 non-adjacent superfractions. The superfractions were acidified with formic acid to a concentration of 1%, followed by vacuum centrifugation. Each superfraction was desalted by StageTipping, dried again by vacuum centrifugation and reconstituted in 5% acetonitrile and 5% formic acid.

### 2.4. Mass Spectrometry Data Acquisition and Processing

Mass spectrometric data were collected using an Orbitrap Fusion Lumos mass spectrometer (Thermo Fisher Scientific), coupled with a Proxeon NanoLC-1200 UHPLC and a FAIMSpro gas-phase fractionation interface [42]. A 100 μm capillary column, packed in-house with 35 cm of C18 beads (Accucore150, 2.6 μm, 150 Å; Thermo Fisher Scientific), was used. Data acquisition was performed over a 90-min gradient. The scan sequence started with a MS1 spectrum acquired in the Orbitrap (resolution: 60,000; scan range: 350–1350 Th; automatic gain control (AGC) target: standard; maximum injection time: auto). The RTS-MS3 scan sequence method was employed to minimize ion interference. MS2 analysis involved collision-induced dissociation (CID) and ion trap analysis (AGC: 2 × 10^4^; maximum injection time: 35 ms; q-value: 0.25; normalized collision energy (NCE): 35%; isolation window: 0.7 Th). The Real-Time Search (RTS) option was utilized with an *S. cerevisiae* yeast database (UniProt, downloaded August 2021), limiting MS3 scans to 2 peptides per protein per fraction. Upon matching a MS2 spectrum, a MS3 spectrum was acquired, capturing multiple MS2 fragment ions using an isolation waveform with multiple frequency notches. MS3 precursors were fragmented by higher-energy collisional dissociation (HCD) and analyzed in the Orbitrap (NCE: 55%; AGC: 1.5 × 10^5^; maximum injection time: 150 ms; resolution: 50,000, sufficient to distinguish TMT isotopologs). A total of 24 RAW files were obtained. For one set of 12 non-adjacent superfractions, a FAIMS compensation voltage (CV) set of −40/−60/−80 V was used, while, for the other 12 superfractions, a CV set of −30/−50/−70 V was applied. Each CV was analyzed using a 1s TopSpeed cycle.

Spectra were converted using MSconvert to mzXML [43]. Database searches included all *S. cerevisiae* entries from UniProt (the same database used for RTS) and all protein sequences in reverse order. Searches were conducted with a 50 ppm precursor ion tolerance and a 0.9 Da product ion tolerance to maximize sensitivity, utilizing Comet database searching and linear discriminant analysis (LDA) [44,45]. TMT tags on lysine residues and peptide N-termini (+304.207 Da) and the carbamidomethylation of cysteines (+57.021 Da) were set as static modifications, while the oxidation of methionine residues (+15.995 Da) was set as a variable modification. Peptide-spectrum matches (PSMs) were adjusted to a 1% false discovery rate (FDR), and further filtering was performed using LDA to achieve a final protein-level FDR of 1% [46,47]. Proteins were quantified by summing reporter ion counts across matching PSMs [48]. Reporter ion intensities were corrected for the isotopic impurities of the TMT reagents, as specified by the manufacturer. The signal-to-noise (S/N) ratios of peptides assigned to each protein were summed and normalized to ensure that the total signal for all proteins in each channel was equal, thereby accounting for equal protein loading (i.e., column normalization). Each protein abundance measurement was then expressed as a percentage of the total, with the summed S/N for that protein across all channels set to 100, providing a relative abundance (RA) measurement. Protein abundance changes were considered statistically significant if they met a fold change cutoff of |log2 ratio| > 0.5 and an uncorrected *p*-value less than 0.05.

## 3. Results and Discussion

### 3.1. Analysis of the Differences in Protein Abundance Between the Strains Lacking Cdc48 Adaptors and the Wild Type Strain Revealed Specific Proteome Level Abundance Changes

To gain a deeper understanding of the cellular role of Cdc48 adaptors, we compared the proteomes of *S. cerevisiae* strains deleted for each of these adaptors with the wild type strain using isobaric-tag-based quantitative proteomics (Figure 1A). We combined, in a single TMTpro18-plex experiment, duplicates of the wild type *S. cerevisiae* strain, single UBX gene deletion strains, *UBX1*(*SHP1*)–*UBX7* and strains deleted for the zinc finger AN1-type domain gene *CUZ1*. Cuz1 is a Cdc48 adaptor, which is essential for protecting budding yeast cells from metalloid toxicity [31]. We wanted to explore the similarities and differences in the proteome among strains deleted in single *UBX* genes and, at the same time, investigate if the deletion of *CUZ1* could induce protein abundance changes in exponentially growing cells not subjected to stress and how these changes were comparable with those induced by the deletion of the UBX family of proteins, which represent a completely different family from *CUZ1*. We cultured yeast cells in a rich medium (YEPD), which we harvested during exponential growth. Their growth was comparable to the wild type strain, except that the *shp1Δ* strain grew 20% slower.

We quantified over 75% of the annotated yeast proteome. Our dataset is composed of 4501 proteins (at a 1% false discovery rate) across all samples. These proteins were identified from 17,620 unique peptides out of a total of 18,453 matched and quantified peptides (Figure 1B). Tables S1 and S2 include the relative abundance measurements of all the proteins and peptides quantified in these experiments, respectively. First, we validated the deletion strains used in the experiment by analyzing the protein levels of the single deleted Cdc48 adaptors. We confirmed their absence in the appropriate deletion strain (Figure S1A). Some residual signal was still observed in the intended knockout strains, which is likely due to interference [49], but this signal was minimal, as expected. We noted variations in the protein levels of Shp1 among the tested strains. Shp1 showed a ~40% increase in both the *cuz1Δ* and *ubx3Δ* strains and ~50% increase in the *ubx5Δ* strain, suggesting that Shp1 could compensate for the loss of these three adaptors. Interestingly, the Shp1 levels were instead ~25% lower in the *ubx7Δ* strain. Additionally, we analyzed the protein levels of Cdc48 itself (Figure S1B) to investigate if any of its adaptors could influence its abundance. The Cdc48 levels were mostly unaltered across the tested strains, except in the *shp1Δ* and in *ubx4Δ* strains, where the Cdc48 levels, respectively, doubled and decreased by ~30%.

**Figure 2:**
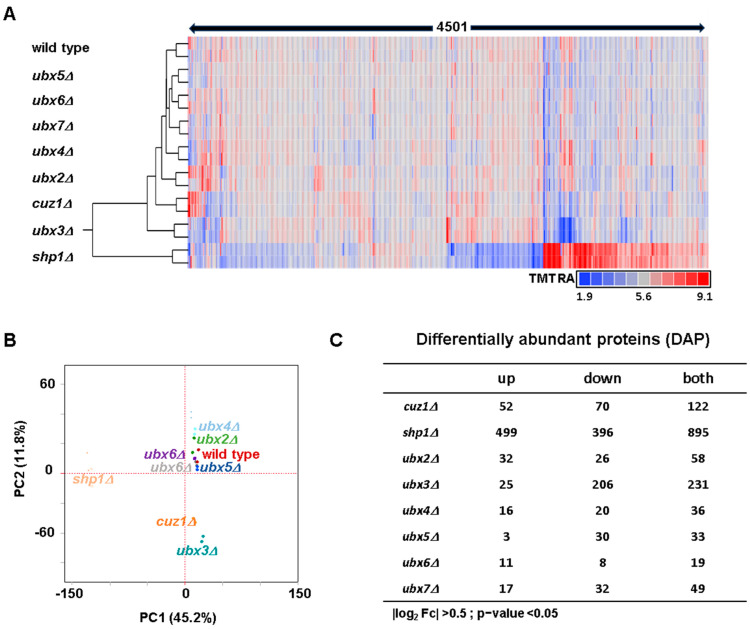
Overview of the dataset. (**A**) Hierarchical clustering of the 4501 proteins across the nine yeast strains used in the proteomic experiment. (**B**) Principal components analysis (PCA) of the samples. (**C**) Summary of the differentially abundant proteins (DAPs) in each strain compared to the wildtype strain.

To explore protein alterations at the global level, we performed both hierarchical clustering analysis (HCA) and principal component analysis (PCA). HCA utilized the Euclidian distance metric and Ward linkage on the relative abundance measurements of the 4501 proteins from this experiment (Figure 2A). Duplicates of the strains clustered together, as anticipated. Interestingly, the *shp1Δ* strain clustered separately from the others, suggesting a more substantial alteration in global proteome abundance. Similarly, PCA confirmed the tight clustering of duplicates in the dataset (Figure 2B). PC1 explained over 45% of the variance (driven by differences in the *shp1Δ* strain), while PC2 explained approximately 12% (driven by differences among strains). PCA also highlighted the similarity between the wild type strain and five adaptor deletion strains (*ubx2Δ*, *ubx4Δ*, *ubx5Δ*, *ubx6Δ* and *ubx7Δ*) and the difference of the *shp1Δ* strain from the others. Furthermore, PCA revealed a certain degree of similarity between the *cuz1Δ* and *ubx3Δ* strains, which clustered closely together. Having validated the strains used in the experiment and observed the expected clustering of duplicates, we proceeded to further analyze the variations in the proteomes of specific deletion strains.

We compared the differences in protein abundance between each strain lacking a specific Cdc48 adaptor and the wild type strain. We defined differentially abundant proteins (DAPs) as those having a >40% increase or decrease (i.e., |log_2_| > 0.5) and an uncorrected *p*-value < 0.05 (Figure 2C). The number of DAPs varied considerably among the tested strains. We list these DAPs, along with their associated abundance measurements, in Table S3. The *shp1Δ* strain exhibited the largest number of DAPs (n = 895) compared to the others. This finding may explain why this strain clustered separately from the others in both HCA and PCA. Following the *shp1Δ* strain, the next-highest number of DAPs were observed in the *cuz1Δ* (n = 122) and *ubx3Δ* (n = 231) strains, which clustered closely together by PCA and separately from the remaining strains. In contrast, the remaining strains (*ubx2Δ*, *ubx4Δ*, *ubx5Δ*, *ubx6D* and *ubx7Δ*) showed fewer proteins changing compared to the wild type (ranging from 19 to 58). These data align well with their clustering proximity to the wild type strain by PCA. Thus, the impact of the analyzed Cdc48 adaptors on the proteome varies significantly. It follows that some of the adaptors for which we detected a lower number of DAPs could act redundantly. Analysis of double- or triple-deletion strains could help decipher their effect on the proteome. In fact, previous studies suggested potential redundancy among *Ubx4*, *Ubx6* and *Ubx7* in regulating ubiquitylated proteins [50].

All analyzed strains exhibited both increasing and decreasing DAPs. Using the list of DAPs, we investigated the similarity and the differences with respect to DAPs across all the eight deletion strains. We found a minimal overlap among DAPs (Figure S2). Most of the DAPs were specific to individual strains with respect to proteins either decreasing (Figure S2A) or increasing (Figure S2B) in abundance, suggesting distinct proteomic changes in the absence of each adaptor. Specifically, no DAPs were shared by all strains, and the number of DAPs shared by multiple strains was relatively low (n = 96 for the DAPs decreasing and 50 for the DAPs increasing). Furthermore, these DAPs tend to be shared between a low number of strains (two or three). In contrast, a considerable number of DAPs were specific to each strain (n = 535 for decreasing DAPs and n = 529 for increasing DAPs). This analysis highlights that the changes at the proteome level induced by the lack of Cdc48 adaptors were specific, indicating that these adaptors likely have specific and distinct cellular functions.

### 3.2. The Upregulation of the Transcription Factor Rpn4 and the Downregulation of Ribosomal Proteins Suggest the Presence of Proteotoxic Stress in the shp1Δ Strain

**Figure 3:**
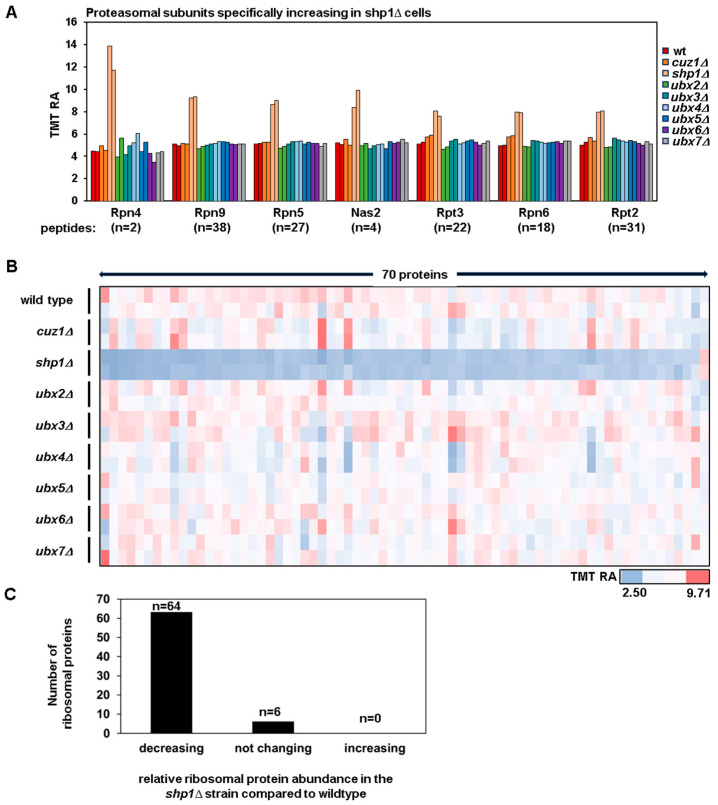
Markers of proteotoxic stress are present in the *shp1Δ* strain but not in the other strains analyzed. (**A**) Bar graph illustrating the TMT relative abundance of the proteasome transcription factor Rpn4 and of the proteasomal subunits (Rpn9, Rpn5, Nas2, Rpt3, Rpn6 and Rpt2) in the strains used in this experiment. The number of peptides detected for each protein is indicated. (**B**) Heatmap showing the relative protein abundance of the ribosome subunits detected in the experiment. (**C**) The number of ribosome subunits and their behavior in the *shp1Δ* strain compared to the wildtype strain is shown.

Cdc48 has an important role in facilitating the delivery of ubiquitylated substrates to the proteasome and to ensure the clearance of protein aggregates. Indeed, the *cdc48* mutant strain accumulates ubiquitylated/misfolded proteins [24]. We hypothesize that the deletion of Cdc48 adaptors could lead to a significant accumulation of ubiquitylated/misfolded proteins, inducing proteotoxic stress. In conditions where the proteasome function is overwhelmed, such as in the presence of proteotoxic stress, the levels of the transcription factor Rpn4 increase to stimulate the production of proteasome subunits, ensuring adequate proteasome function [51,52]. We analyzed the protein levels of Rpn4 and observed a three-fold increase in the *shp1Δ* strain (Figure 3A), while, in all other deletion strains, the Rpn4 levels were comparable to those in the wild type strain. Consistent with Rpn4’s role in inducing the transcription of proteasome subunits, we also observed an increase in protein levels of multiple proteasome subunits, such as Rpn5, Rpn9, Nas2, Rpt3, Rpn6 and Rpt2 (Figure 3A).

Previously, the proteotoxic stress response in *S. cerevisiae* [53] was shown to be characterized by a reduction in ribosomal proteins. We indeed analyzed the levels of these proteins in our dataset. Remarkably, we found that they decreased exclusively in the *shp1Δ* strain (Figure 3B). We detected 70 ribosomal proteins in the experiment, and 64 showed decreased protein levels in the *shp1Δ* strain, while 6 were unaltered compared to the wild type (Figure 3C). We have included the names and quantitative information of these 70 ribosomal proteins in Table S4. Additionally, alongside the decrease in ribosomal subunits, we observed a reduction in the proteins responsible for ribosomal biogenesis, such as Alb1, Bms1 and Brx1.

The increased levels of Rpn4 and proteasomal subunits, along with the concomitant decrease in ribosomal proteins, strongly suggests that the *shp1Δ* strain experienced proteotoxic stress. This observation was consistent with this strain growing slower compared to the other strains. Since Shp1 has been identified in multiple studies as the major Cdc48 adaptor involved in delivering proteins to the proteasome [54,55], it is likely that, in the absence of *SHP1*, cells accumulated ubiquitylated proteins, thereby triggering a proteotoxic stress response.

### 3.3. A Specific Subset of Mitochondrial Proteins Is Downregulated in the ubx3Δ and cuz1Δ Strains

Hierarchical clustering analysis revealed a distinct group of proteins that were strongly downregulated in the *ubx3Δ* strain but not in the other strains (Figure 2A). We analyzed the 206 differentially abundant proteins (DAPs) that showed a decrease specifically in the *ubx3Δ* strain. We found that 123 were mitochondrial proteins.

In our dataset, we quantified a total of 442 mitochondrial proteins (Figure S3A) and observed a broad decrease in their levels, particularly pronounced in the *ubx3Δ* strain. Although some mitochondrial proteins (n = 35) also showed reduced levels in the *cuz1Δ* strain, the effect was less pronounced compared to the *ubx3Δ* strain. Therefore, we focused our analysis on the mitochondrial proteins decreasing in the *ubx3Δ* strain (Figure S3B). We found that a significant portion of the mitochondrial proteins decreasing in this strain were mitochondrial ribosome subunits (n = 43). Furthermore, numerous proteins were part of mitochondrial protein complexes (n = 31), including the Cytochrome b-c1 complex, the Cytochrome C oxidase complex (n = 17) and the ATP synthase complex (n = 14) (Figure S3B), as well as metabolic enzymes (n = 15). The remaining mitochondrial proteins that showed decreased abundance included a miscellaneous group (n = 33) such as transporters, outer membrane proteins and proteins involved in RNA metabolism. Moreover, while Cdc48 has been implicated previously in mitochondrial regulation [22,56,57,58], our analysis suggested that *Ubx3* was crucial for maintaining the wild type levels of many mitochondrial proteins, a function not associated previously with *Ubx3*.

### 3.4. Examples of Proteins That Change Specifically in Cdc48 Adaptor Protein Deletion Strains

Our analysis revealed a very low overlap in DAPs among adaptor deleted strains, with most proteins uniquely changing in one strain (Figure S2). This finding suggests that these adaptors are associated with specific ubiquitylated proteins or binding partners and are likely to perform distinct cellular functions. Among these specific proteins, we highlighted the presence of kinases, such as Chk1 and Tda1. Chk1, a highly conserved kinase essential for the DNA damage response [59], showed specific strong downregulation specifically in the *ubx7Δ* strain (Figure S4A), implying a potential role for this adaptor in the DNA damage response of *S. cerevisiae*. Tda1, a kinase involved in glucose metabolism [60], exhibited lower protein levels in the *shp1Δ* strain and higher levels in the *cuz1Δ* strain (Figure S4A), suggesting a possible role for these two adaptors in sugar metabolism.

A notable finding was the differential regulation of components belonging to the UPS, such as the deubiquitylating enzymes (Figure S4B), for which the protein levels were up- or downregulated in specific strains. The *shp1Δ* strain showed an increase in Ubp7 and a decrease in Ubp10 abundance. Conversely, Ubp2 abundance decreased strongly only in the *ubx2Δ* strain, whereas Ubp16 showed increased abundance exclusively in the *cuz1Δ* strain (Figure S4B). As deubiquitylating enzymes remove ubiquitin from proteins, thereby rescuing them from degradation, the alterations in individual protein abundance in these strains could contribute to some of the changes in the proteome that we observed. With these few examples in mind, we suspect that the 1400 proteins that we determined to be differentially abundant in the Cdc48 adaptor deletion strains will comprise a valuable resource to help us fully understand the specific cellular functions regulated by these adaptors.

## 4. Conclusions and Limitations

Here, we presented the first quantitative proteome profiling of eight *S. cerevisiae* deletion strains of Cdc48 adaptors compared to the wild type strain. We quantified 4501 proteins, accounting for approximatively 75% of the yeast proteome. Our analysis revealed the distinct impacts of these adaptors on the proteome; for example, *SHP1* and *CUZ1* deletions affect the abundance of hundreds of proteins, whereas the deletion of others such as *UBX4*, *UBX5*, *UBX6* and *UBX7* affect fewer than fifty proteins each. Remarkably, each deleted strain exhibits a unique set of differentially abundant proteins, reflecting the specificity of these adaptors.

Our proteome analysis serves as a resource and as a starting point for the many researchers interested in the Ubiquitin Proteasome System—in particular, cellular processes controlled by Cdc48 or in mechanisms regulating a specific protein of interest. As such, we recommend conducting orthogonal and targeted experiments. It is crucial to perform orthogonal validation and manually verify the peptide spectra for certain proteins of interest, as false spectral assignments can be made for data of low spectral quality. We recognize that yeast deletion strains frequently accumulate adaptive genetic alterations that may compromise proteomic alterations. To minimize this issue, we tried to dilute them by using a population of yeast streaked from across an agar plate rather than single colonies.

A limitation of our study is that it profiles only a single proteomic experiment. This means that it is possible that the abundance differences of some proteins may not be reproducible, especially if detected with a single peptide or if characterized by a small fold change. We were limited to biological duplicates for each condition due to the upper limit of multiplexing capacity at this time (18 channels).

Another limitation of our study is that we utilized consensus protein sequences for building our dataset, thereby excluding proteoforms. Similarly, we did not consider post-translational modifications (e.g., phosphorylation and ubiquitylation, which would have required upstream enrichment) that could also differ between these strains. This post-translational modification analysis could reveal other differences among the strains deleted in Cdc48 adaptor genes. Furthermore, as some of these adaptors have been shown to act redundantly, a combination of their deletions could reveal other differentially abundant proteins.

Nonetheless, our analysis revealed interesting insights into how single deletions of Cdc48 adaptors alter the proteome. We highlighted that only the *SHP1* deletion strain shows markers of proteotoxic stress, suggesting potential overload of the UPS in this strain. Furthermore, we observed a specific decrease in mitochondrial proteins in the absence of *UBX3*, suggesting that this adaptor could control mitochondrial function. In summary, our dataset included approximatively 1400 differentially abundant proteins in the Cdc48 adaptor deletion strains, thereby providing a valuable resource for studying these adaptors and for better understanding the cellular processes regulated by these adaptors and by Cdc48 itself.

## Data Availability

RAW files have been deposited to the ProteomeXchange Consortium via the PRIDE [61] partner repository with the dataset identifier PXD055137.

## Supplementary Materials

The following supporting information can be downloaded at https://www.mdpi.com/article/10.3390/proteomes12040031/s1: Figure S1: Relative abundance plots verify deletion strains and highlight differences in Cdc48 protein abundance; Figure S2: Minimal overlap of the differentially abundant proteins among the deletion strains investigated herein; Figure S3: A specific set of mitochondrial proteins is decreasing at the protein level in the ubx3D strain; Figure S4: Examples of proteins that change specifically in one of the Cdc48 adaptor protein deletion strains. Table S1: Proteins quantified in the experiment. Columns include Protein ID, Gene symbol, Description, Number of Peptides assigned to a given protein, and 18 columns of TMT signal-to-noise values that have been scaled to 100 across all channels. Table S2: Peptides quantified in the experiment. Columns include UniProt protein ID, gene symbol, protein description, redundancy, whether peptide is a unique or razor peptide, peptide sequence, and TMT signal-to-noise for all channels; Table S3: List of the differentially abundant proteins (DAPs) in each deletion strain compared to the wildtype. Columns include Protein ID, Gene Symbol, Description, Number of Peptides, log2 Fc, and log10 *p*-value for both proteins decreasing and increasing. Data for each deletion strain can be found in the appropriately labeled tab; Table S4: List of the ribosomal proteins detected in the experiment. Columns include Protein ID, Gene Symbol, Description, Number of Peptides, and the 18 TMT signal-to noise values that have been scaled to 100.
