# Supplementary material for: Comparative Proteome-Wide Abundance Profiling of Yeast Strains Deleted for Cdc48 Adaptors
Source: Proteomes. 2024 Oct 30;12(4):31. doi: 10.3390/proteomes12040031 (PMC11587464; doi:10.3390/proteomes12040031)
Supplement: Supplementary file 1 [file proteomes-12-00031-s001.zip › SuppMet-proteomes-3199095 .pdf]

Supplemental material for:

Comparative proteome-wide abundance profiling of yeast strains deleted for Cdc48 adaptors

Valentina Rossio * and Joao A Paulo *

Department of Cell Biology, Harvard Medical School, Boston, MA 02115, USA.

Figure S1: Relative abundance plots verify deletion strains and highlight differences in Cdc48 protein abundance.

Figure S2: Minimal overlap of the differentially abundant proteins among the deletion strains investigated herein.

Figure S3: A specific set of mitochondrial proteins is decreasing at the protein level in the *ubx3D* strain.

Figure S4: Examples of proteins that specifically change in one of the Cdc48 adaptor protein deletion strains.

Table S1: Proteins quantified in the experiment. Columns include: Protein ID, Gene symbol, Description, Number of Peptides assigned to a given protein, and 18 columns of TMT signal-to-noise values that have been scaled to 100 across all channels.

Table S2: Peptides quantified in the experiment. Columns include: Uniprot protein ID, gene symbol, protein description, redundancy, whether peptide is a unique or razor peptide, peptide sequence, and TMT signal-to-noise for all channels.

Table S3: List of the differentially abundant proteins (DAPs) in each deletion strain compared to wild type. Columns include: Protein ID, Gene Symbol, Description, Number of Peptides, log_2 Fc and log_{10} p-value for both proteins decreasing and increasing. Data for each deletion strain can be found in the appropriately-labeled tab.

Table S4: List of the ribosomal proteins detected in the experiment. Columns include: Protein ID, Gene Symbol, Description, Number of Peptides and the 18 TMT signal-to noise value that has been scaled to 100.

Figure S1:

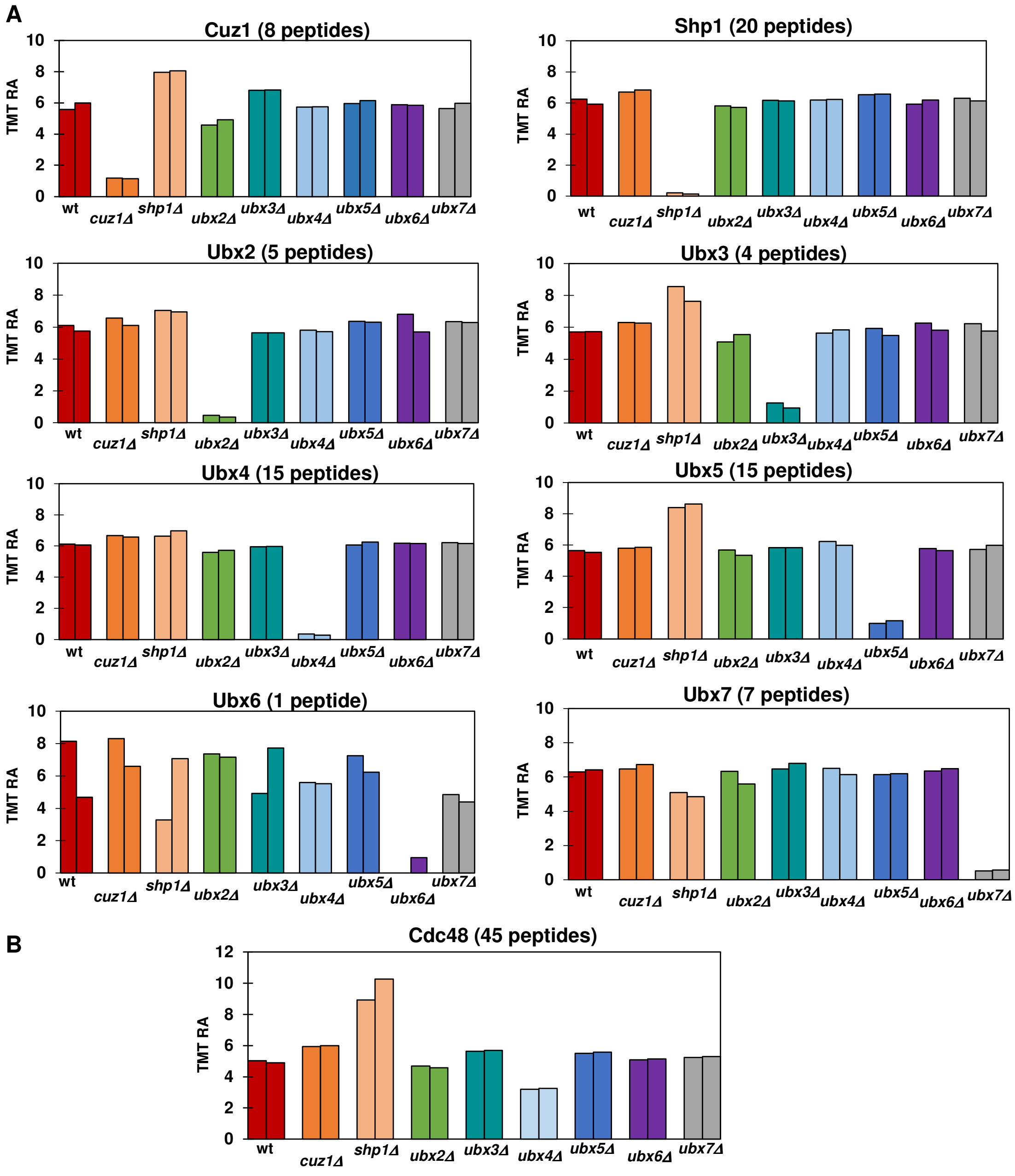

Figure S1: **Relative abundance plots verify deletion strains and highlight differences in Cdc48 protein abundance.** Analysis of the TMT relative protein abundance (TMT RA) of both (**A**) the adaptors of Cdc48 deleted in this study to confirm the correct genotype and of (**B**) Cdc48 itself. Bar graphs illustrating the TMT RA for the indicated deleted proteins in each deletion strain. The number of peptides detected for each protein is indicated.

Figure S2:

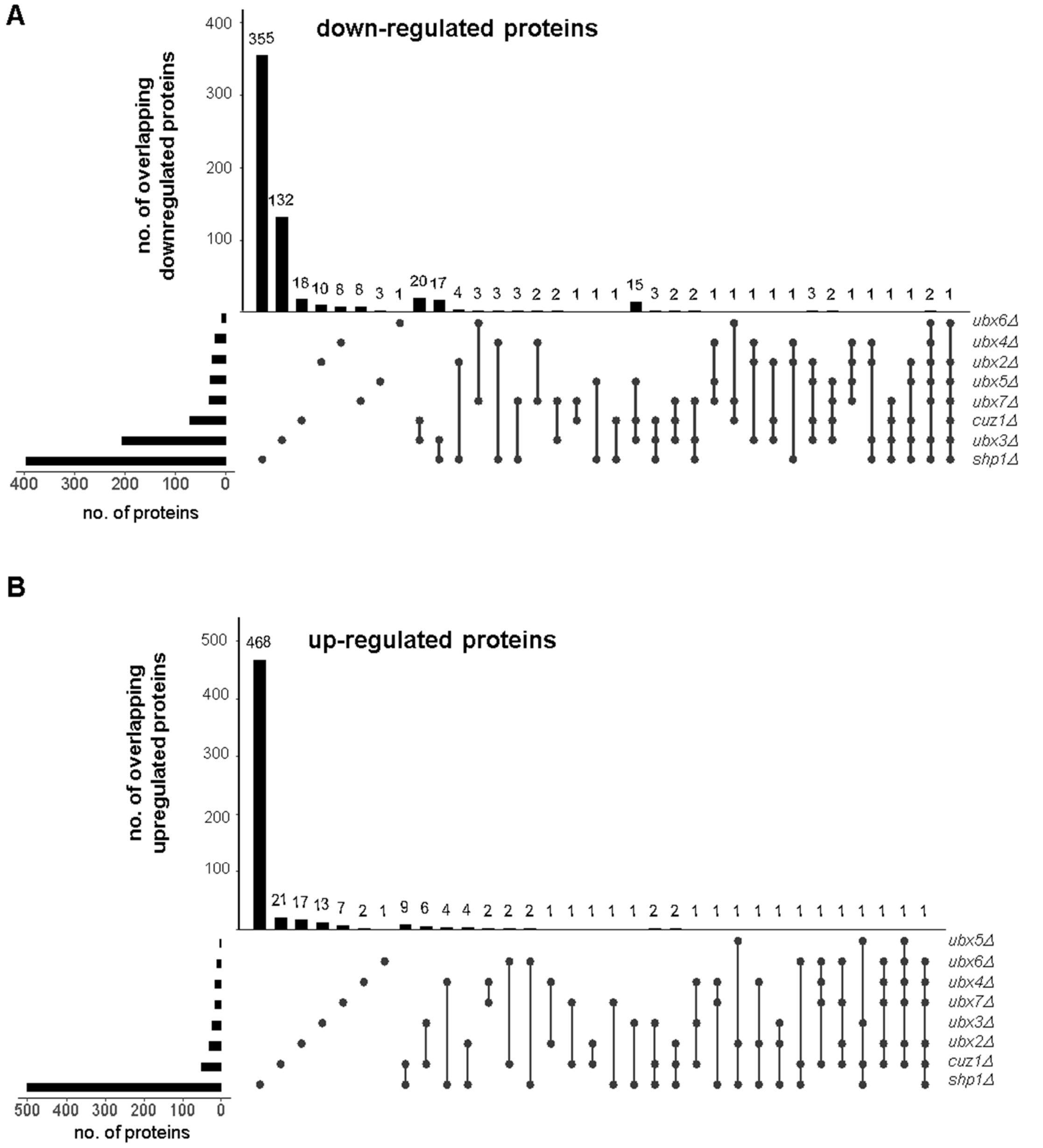

Figure S2: Minimal overlap of the differentially abundant proteins among the deletion strains investigated herein. UpSet plots illustrating both the overlap of **(A)** down- and **(B)** up-regulated proteins with respect to the different Cdc48 adaptor protein knockout strains and the wildtype strain.

Figure S3:

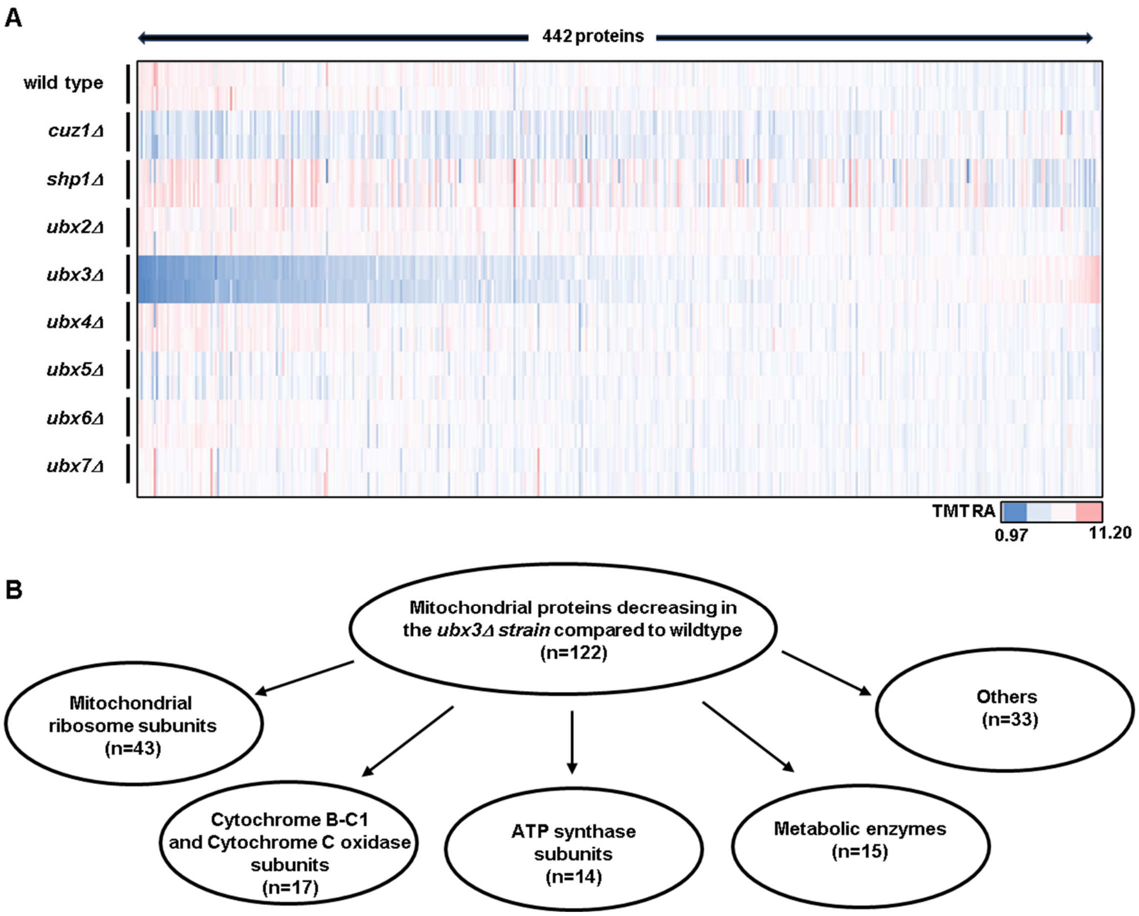

Figure S3: A specific set of mitochondrial proteins is decreasing at the protein level in the *ubx3D* strain. (A) Heatmap showing the TMT relative protein abundance of all the mitochondrial proteins quantified in this experiment. **(B)** Mitochondrial proteins decreasing at the protein level in the *ubx3D* strain compared to wildtype strain. The number of mitochondrial proteins in each category is indicated in parentheses.

Figure S4:

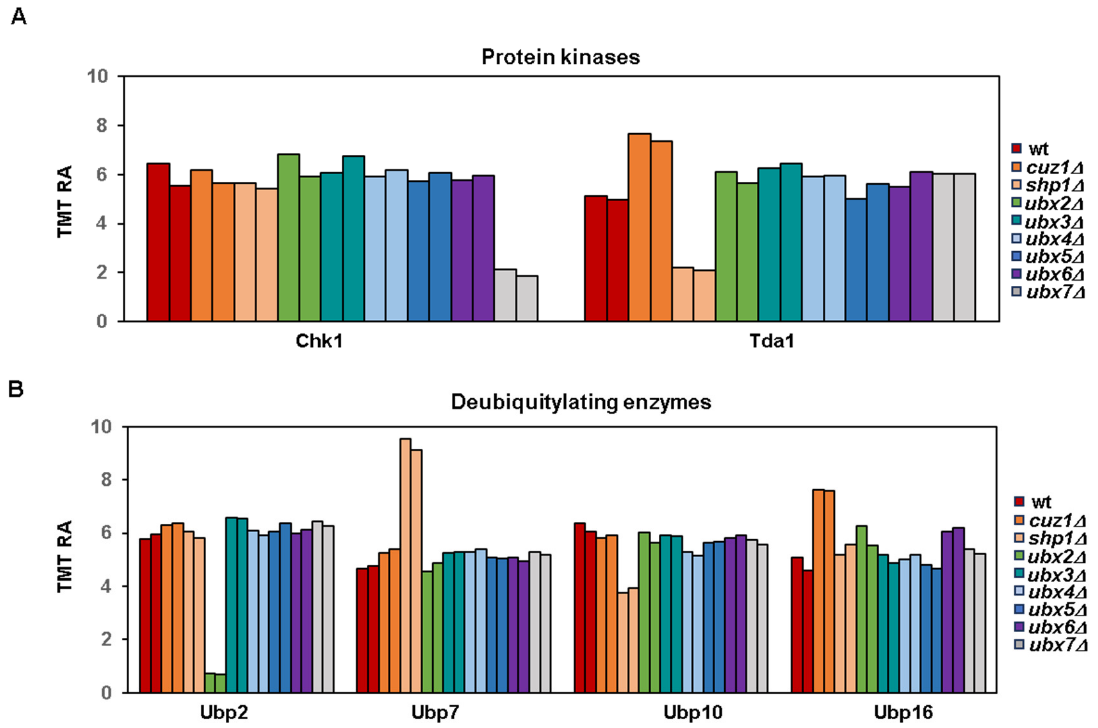

Figure S4: Examples of proteins that specifically change in one of the Cdc48 adaptor protein deletion strains. Bar graph illustrating the TMT relative protein abundance (TMT RA) **(A)** of the kinases Chk1 and Tda1 **(B)** of the four deubiquitylating enzymes: Ubp2, Ubp7, Ubp10 and Ubp16.

Table S1: Proteins quantified in the experiment. Columns include: Protein ID, Gene symbol, Description, Number of Peptides assigned to a given protein, and 18 columns of TMT signal-to-noise values that have been scaled to 100 across all channels.

Table S2: Peptides quantified in the experiment. Columns include: Uniprot protein ID, gene symbol, protein description, redundancy, whether peptide is a unique or razor peptide, peptide sequence, and TMT signal-to-noise for all channels.

Table S3: List of the differentially abundant proteins (DAPs) in each deletion strain compared to wild type. Columns include: Protein ID, Gene Symbol, Description, Number of Peptides, $\log_2$ Fc and $\log_{10}$ p-value for both proteins decreasing and increasing. Data for each deletion strain can be found in the appropriately-labeled tab.

Table S4: List of the ribosomal proteins detected in the experiment. Columns include: Protein ID, Gene Symbol, Description, Number of Peptides and the 18 TMT signal-to noise value that has been scaled to 100.
